# Excessive Bacteria in Milk Leads to Heavy Fines and Jail Sentence

**Published:** 1913-11

**Authors:** 


### Excessive Bacteria in Milk Leads to Heavy Fines and Jail Sentence.

Notices of judgment, just issued by the Department of Agriculture, state that fines have been imposed on several shippers of adulterated milk and cream.

John Fisher, Newport, Kentucky, was fined $100, and sentenced to 60 days imprisonment for shipment from Kentucky into Ohio of milk and cream alleged to be adulterated. Some of the product was labeled on the cap: "Guaranteed Pure Milk. Please put bottles out every day."

Twenty-nine samples of milk, analyzed separately, were either skimmed or skimmed and watered, and all contained visible sediment of dirt and filth. Five samples of alleged cream were found to contain only 12.5 per cent of milk fat and to be artifically colored with annatto.

Adulteration of the product was alleged because the milk and cream consisted in part of filthy and decomposed animal and vegetable substance, since they contained an excessive number of bacteria, including members of a group known as "B. coli."

The 60 days' jail sentence was suspended on condition that the defendant keep out of the milk business.

The Lehigh Valley Railroad Co. was charged with having sold on its dining car so-called "cream," which contained excessive numbers of objectionable and unhealthful bacteria, and was fined $25 and costs, since the transaction involved the shipment from Pennsylvania into New Jersey of said milk and cream. Adulteration was alleged for the reason that said products consisted in whole or in part of a filthy and putrid animal and vegetable substance.

Ralph S. Richardson, of Pierceville, Indiana, was fined $25 and costs, for shipment into Ohio of a quantity of milk alleged to be adulterated. The adulteration charged in this case was that water had been added to the milk. The court imposed a like fine on Hubert E. Nead, also of Pierceville, Indiana, upon similar charge.

---

Little Johnny—Dad, there's a girl at our school whom we call "Postscript."

Dad—"Postscript!" Whatever do you call her "Postscript" for?

Little Johnny—'Cos her name is Adeline Moore!—*Tit-Bits.*

The employment of narcosis in a case of "stiff and painful shoulder" may reveal a cause not otherwise ascertainable, *e. g.,* subluxation.—*American Journal of Surgery.*

